# Autism Spectrum Disorders: Prenatal Genetic Testing and Abortion Decision-Making among Taiwanese Mothers of Affected Children

**DOI:** 10.3390/ijerph17020476

**Published:** 2020-01-11

**Authors:** Wei-Ju Chen, Shixi Zhao, Tse-Yang Huang, Oi-Man Kwok, Lei-Shih Chen

**Affiliations:** 1 Psychology Department, The University of Texas Permian Basin, Odessa, TX 79762, USA; chen_w@utpb.edu; 2 Department of Health, Exercise, and Sports Sciences, University of New Mexico, Albuquerque, NM 87131, USA; shixizhao@unm.edu; 3 Department of Special Education, National Tsing Hua University, Hsinchu 30013, Taiwan; tyhuang@mail.nd.nthu.edu.tw; 4 Department of Educational Psychology, Texas A&M University, College Station, TX 77843, USA; omkwok@tamu.edu; 5 Department of Health and Kinesiology, Texas A&M University, College Station, TX 77843, USA;

**Keywords:** abortion, autism spectrum disorders, mothers, prenatal genetic testing, structural equation modeling, Taiwan, termination of pregnancy

## Abstract

With the rapid growing rate of autism spectrum disorders (ASDs), prenatal genetic testing (PGT) has been offered to detect various genomic disorders, including ASD, in Taiwan. However, disparities exist in this area, as there is limited research on factors associated with PGT utilization and relevant decision-making that may guide the regulations and ethical guidelines for culturally appropriate PGT services in Taiwan. This study proposed a comprehensively integrated theoretical framework for examining the intention to undergo PGT to detect ASD susceptibility genes and subsequent abortion decision-making among Taiwanese mothers of children affected by ASD. Survey data from 333 mothers of children with ASD in 236 elementary schools with special education services in Taiwan were collected and analyzed using structural equation modeling. Approximately two-thirds of the participants (66.6%) would undergo PGT to detect ASD susceptibility genes; more than half (53.1%) would terminate the hypothetically ASD-affected pregnancy. Abortion intention was associated with age, religion, attitudes toward PGT for detecting ASD susceptibility genes, and willingness to undergo such PGT. This study explores the potential impacts of PGT on Taiwanese society, and the findings are applicable to countries heavily influenced by Chinese culture, areas with Asian immigrants, and Western countries with such PGT services and/or research available.

## 1. Introduction

Autism spectrum disorders (ASDs) are neurodevelopmental disorders characterized by challenges or impairments in language, communication, and social interaction [1,2]. In Taiwan, ASDs are the fastest growing developmental disorder [3]. Comparing the data from the Ministry of Health and Welfare between 2005 and 2016, Taiwanese individuals with ASDs increased by 250% [4]. Such an increase is attributed to changes in the diagnostic criteria, increased awareness of ASDs in the general public, improved access to ASD-related services, and lower discrimination against families affected by ASDs [5,6]. Research studies on twins, relatives, and rare syndromes associated with ASDs [2,7] suggested the critical role and contribution of genetics in the etiology of ASD. The heritability of ASD is high with estimations of 64% to 91% [8], and over 200 genes related to ASD have been identified [9].

In Taiwan, prenatal genetic testing (PGT) via amniocentesis has been clinically offered to pregnant women regardless of ASD family history during gestation weeks 16 to 20 by obstetricians to detect a fetus’ risks for various genetic and genomic disorders, including ASDs [10,11,12,13]. Chromosomal microarray (CMA) has promising clinical validity [14], higher detection rates [15,16], and better diagnostic yields [14,17] compared to other testing methods (e.g., karyotype). Thus, CMA is the most common and first-tier method in prenatal settings used to detect inherited or de novo copy number variants associated with ASD [2,7,13,15,17]. The National Health Insurance, covering 99.6% of Taiwanese residents [18], does not cover this PGT. Parents have to pay for this test themselves, which costs approximately 18,000 New Taiwan Dollars (~US$592) [10,12,13].

There are benefits to PGT for detecting ASD susceptibility genes within fetuses, as it can provide information to parents regarding their unborn children’s risks for ASDs and facilitate informed decision-making. Results of PGT may allow these parents to plan for early intervention and/or preparation for their children who may be affected by ASDs [19,20]. Nevertheless, such PGT has limitations. Specifically, ASDs are complex heterogeneous disorders with multifactorial etiologies [21]. Although the role of genetics in ASD etiology has been extensively studied, causative effects of many genetic variants remain unknown, and reduced/incomplete penetrance and variable expressivity are testing concerns [21,22]. Moreover, while CMA has higher diagnostic yields [14,17] compared to other testing methods, the diagnostic yields (9.3% to 24.0%) [23] and overall detection rate for ASD (24.4%) [24] are moderate. Furthermore, the results of PGT may be difficult to interpret and explain by obstetricians [13,19].

In addition to the testing restrictions, PGT for detecting ASD susceptibility genes raises ethical, legal, and social concerns, particularly with the topic of abortion [13,19,20]. Our past qualitative study [13] suggested that two-thirds of the 39 participating Taiwanese parents of children with ASDs would undergo PGT for detecting ASD susceptibility genes within their fetuses. The termination of affected pregnancies is those parents’ primary motive for undergoing such PGT. Due to the limitations and exploratory nature of qualitative research, a quantitative study with a larger sample and advanced statistical analyses is needed to further understand this topic [25]. Moreover, there is limited research on factors associated with the intention to utilize PGT and relevant subsequent decision-making that may be used to guide the establishment of policies, regulations, and ethical guidelines for culturally appropriate PGT services in Taiwan; therefore, more research, particularly among underserved populations, is essential to address such disparities and gaps in this area.

To the best of our knowledge, this is the first study that examined PGT for detecting ASD susceptibility genes and abortion decision-making among Taiwanese mothers of children diagnosed with ASDs. To capture comprehensive aspects of the intention to undergo PGT for detecting ASD susceptibility genes and address the lack of theory-based models in the literature, we proposed and tested an integrated theoretical framework. This framework was developed based on the health belief model [26], theory of planned behavior [27], and social cognitive theory [28], which have been used in assessing individuals’ behavior regarding genetic testing/screening [29,30,31]. We aimed to advance the study of genomic translation among underserved/under-researched populations affected by ASDs and provide insights into better genomic practice related to PGT. Understanding mothers’ views on PGT and subsequent abortion decision-making may assist obstetricians and other health professionals in providing appropriate prenatal care, education, and counseling [32]. 

## 2. Materials and Methods 

### 2.1. Participants

Participants (443 biological mothers and fathers of children with ASD) were recruited from 236 public elementary schools that provide special education services in Taiwan. This particular group was selected due to the increased recurrence risks of having another child with ASD [33]. Mothers of existing children diagnosed with ASD may be most affected by the PGT for detecting ASD susceptibility genes within fetuses because they are often the child-bearers and decision-makers for PGT and the termination of pregnancies [33,34]. This study, therefore, focused on the 333 mothers of children diagnosed with ASD. 

The mean age of the participants was 38.93 years (*SD* = 4.88). The mean age of their children with ASD was 9.61 years (*SD* = 4.88), and the average time since their first ASD diagnosis was 61.82 months (*SD* = 32.15). Most of the participants were married (90.1%) and held religious beliefs (77.6%). Almost one-third of the participating mothers (32.2%) has received a bachelor’s degree or above, 29.1% held an associate’s degree, and 38.7% had an education level of high school or less. Over one-third of the participants (35.7%) had family histories of ASD. Detailed descriptive statistics for the sample characteristics are shown in Table 1. 

### 2.2. Data Collection Procedure

The present study is part of a large research project that assessed genomic issues related to ASD in Taiwan, and the study protocol was approved by the Institutional Review Board at Texas A&M University and the Research and Development Office at the National Tsing Hua University in Taiwan. The main recruitment took place in Hsinchu City and County, Taoyuan County, and Miaoli County, in which the Department of Education in Taiwan provided the research team with a list of all public elementary schools with special education services in these cities and counties. The special education teachers in these areas helped recruit parents of children with ASD by administering paper-and-pencil surveys as this was the participants and special education teachers’ preferred method. Additional surveys were mailed to the prospective participants in Taipei City, New Taipei City, Taichung City, Tainan City, Kaohsiung City, Yilan County, Chiayi City, Chiayi County, and Yunlin County to recruit more parents. Informed consent was obtained from the participating parents. The incentives for completing the survey included: An entry into a draw with eight NT$3000 (~US$99) vouchers, 20 NT$2,000 (~US$66) vouchers, and 200 NT$1000 (~US$33) vouchers. The response rate was 52.3% (451 of the 862 surveys were returned). Eight surveys were invalid due to incompletion, unknown responses, or duplication. 

### 2.3. Measures

Based on past research [19,29,30,31,35,37,38,39,40,41,42,43,44], we developed a survey in Mandarin to examine factors associated with the mothers’ intention to undergo PGT for detecting ASD susceptibility genes within their fetuses (“If you are pregnant, how likely are you to undergo PGT for detecting ASD susceptibility genes?”) and their abortion decision-making in a hypothetical scenario (“If testing results indicate that you might have a fetus with ASD, what would be your choice?”). To account for the comprehensive aspects of such intention and abortion decision-making, the factors assessed were based on our proposed integrated theoretical framework consisting of the health belief model [26], theory of planned behavior [27], and social cognitive theory [28]. Specifically, mothers’ intention to terminate the affected pregnancy is associated with their intention to undergo PGT for ASD. Both intentions are linked to mothers’ attitudes toward PGT for ASD, which is related to their perception of the severity of ASD, recurrence risks of having another child with ASD, and benefits of PGT for ASD. Mothers’ likelihood of undergoing such PGT is also correlated to their self-efficacy and subjective norms regarding undergoing PGT for detecting ASD susceptibility genes. In addition, self-efficacy is associated with mothers’ perceived barriers to undergoing PGT. Table 2 shows the definitions of the constructs in the survey, the number of items in each construct, and example questions. The covariates included socio-demographic variables, knowledge of genetic testing for ASD, and perceived genetic ASD etiology. 

Past research suggests that the lay public in Taiwan has low genetic literacy [13,38]. To ensure that our participants could understand the survey questions, to accurately examine their knowledge of PGT, and to reflect realistic situations in prenatal settings in Taiwan (e.g., short doctor’s appointment time [45] and limited genetic counseling from genetic professionals [46]), we used simple language without providing extensive education of PGT for detecting ASD susceptibility genes in the survey. Physicians, pediatricians, social behavioral scientists, and special education professionals reviewed our survey for content validity. The survey was then pilot tested with a small sample of parents of children with ASD. 

### 2.4. Statistical Analysis

Descriptive statistics were conducted for all variables. To examine the data reliability and validity, survey items were analyzed using Cronbach’s alpha (α) and confirmatory factor analysis (CFA) with SPSS 22.0 (IBM, Armonk, New York, U.S.) and Mplus 8.0 (Muthén & Muthén, Los Angeles, California, U.S.), respectively. All constructs were psychometrically sound (shown in Table 2). Furthermore, using Mplus 8.0, structural equation modeling (SEM) with weighted least square means and variance adjusted estimation was utilized to test the model fit. Chi-square statistics, root mean square error of approximation (RMSEA; a cutoff of < 0.10), comparative fit index (CFI; a cutoff of > 0.90), and weighted root mean square residual (WRMR; a cutoff of < 1.0) were reported for the model fit [47,48].

## 3. Results 

### 3.1. Psychosocial Factors Associated with PGT for Detecting ASD Susceptibility Genes and Abortion Decision-Making 

As shown in Table 2, the mothers in our sample reported (1) high perceived severity of ASD, (2) positive perceived benefits of PGT for detecting ASD susceptibility genes in general and for the purpose of family planning, (3) strong subjective norms (associated with professionals, family members, and others), (4) high self-confidence in undergoing PGT, and (5) favorable attitudes toward PGT for detecting ASD susceptibility genes within their fetuses. Moreover, 66.6% of the mothers stated that they were either likely or very likely to undergo PGT, and 53.1% would terminate the pregnancy if ASD susceptibility genes were found within the fetuses. 

### 3.2. SEM Findings 

**Figure 1:**
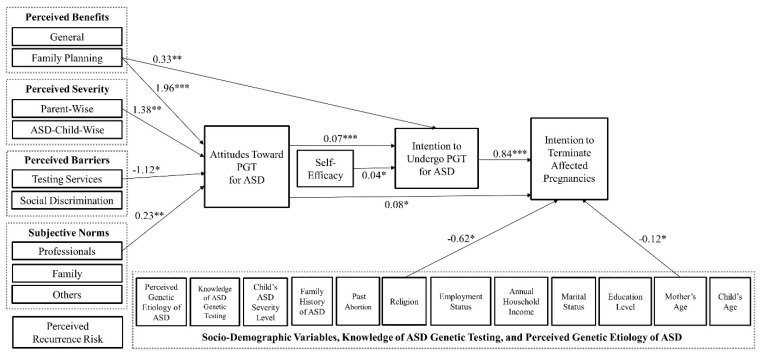
Structural model: factors associated with intention to undergo prenatal genetic testing (PGT) for detecting autism spectrum disorders (ASD) susceptibility genes and to terminate affected pregnancies among Taiwanese mothers of children with ASD in our sample, controlling for socio-demographic variables, knowledge, and perceived genetic etiology of ASD. Due to the multicollinearity related to the child’s age, time since ASD diagnosis was not included in this final model. Only the statistically significant associations (represented using solid lines) are presented in this figure. * *p* < 0.05. ** *p* < 0.01. *** *p* < 0.001.

SEM results (Figure 1) suggested a good model fit between the proposed theoretical model and the survey data after controlling for the covariates (χ^2^ = 23.84, df = 17, *p* = 0.12, RMSEA = 0.039, CFI = 0.95, WRMR = 0.47). Our model explained 63.0% of the variance in abortion decision-making among the mothers of children with ASD who completed the survey. Participants’ intention to terminate ASD-affected pregnancies was significantly and positively related to their attitudes (B = 0.08; *p* < 0.05) and intention to undergo PGT for detecting ASD susceptibility genes within their fetuses (B = 0.84; *p* < 0.001). Mothers’ attitudes were positively associated with the perceived family planning-related benefits (B = 1.96; *p* < 0.001), perceived severity regarding the impacts of ASD on their lives (B = 1.38; *p* < 0.01), and perceived influences of professionals, including physicians, other non-physician health professionals, and school teachers of children with ASD (B = 0.23; *p* < 0.01). Furthermore, participating mothers’ attitudes were negatively correlated with their perceived barriers associated with the testing services (B = −1.12; *p* < 0.05). 

Participants’ intention to undergo PGT to detect ASD susceptibility genes within their fetuses was associated with their attitudes (B = 0.07; *p* < 0.001), self-efficacy (B = 0.04; *p* < 0.05), and perceived family planning-related benefits in undergoing such PGT (B = 0.33, *p* < 0.01) in a positive direction. Among all covariates, the mother’s age had a significant and negative association with their abortion intention (B = −0.12; *p* < 0.01). Mothers who had religious beliefs in this study reported less willingness to abort pregnancies affected by ASD compared to mothers who had no religious beliefs (B = −0.62; *p* < 0.05).

## 4. Discussion

The present study is the first to utilize an integrated theoretical framework to examine the psychosocial factors associated with the intention to undergo PGT for detecting ASD susceptibility genes within the fetuses and subsequent abortion decision-making among mothers of existing children with ASD in Taiwan. A good model fit was found showing that our survey data supported the proposed framework. In particular, the mothers’ intention to terminate ASD-affected pregnancies was associated with favorable attitudes toward PGT for detecting ASD susceptibility genes and a higher intention to undergo such PGT. 

Our data suggested that participants generally favored PGT for detecting ASD susceptibility genes. Slightly over half of the sample would choose to terminate the pregnancy if ASD susceptibility genes were found within the fetus(es). These percentages were higher than a previous study conducted in the United States (U.S.). Specifically, in a qualitative study with 42 U.S. parents of children with ASD as a sample, 57.1% of the participants indicated an interest in undergoing PGT for detecting ASD susceptibility genes for their fetuses. Among those parents, only 19.4% reported that they would terminate the affected pregnancies based on the results of the PGT [19]. Nevertheless, our findings are consistent with the past research with Chinese Americans, in which participants expressed interests in PGT for psychological disorders, such as ASD, and would terminate the affected pregnancies [20]. Moreover, in our previous qualitative study, abortion is the most prominent motivation for Taiwanese parents of children with ASD to undergo PGT for detecting ASD susceptibility genes. The potential explanation could be that in Taiwan, having a child with mental, intellectual, or development disability is associated with cultural stigma and disgrace for the family, which may impact mothers’ perceptions regarding PGT and abortion [13,50]. Moreover, the Genetic Health Act [51] in Taiwan has made abortion easier for pregnant women. Women may have an abortion if they believe that the pregnancy may affect their mental health, physical health, or family life. Other factors, such as family burdens, psychological hardship, and lack of adequate resources and social welfare to support families of children with ASD, might provide additional interpretation as well [13,20,50,52].

With the rapidly growing rate of ASD [3,4], the findings of this study may bring attention to the ethical, legal, and social issues regarding PGT for detecting ASD susceptibility genes in Taiwan. Particularly, there is a lack of policies and guidelines that regulate and monitor the delivery and quality of such PGT [13]. Without clearly established ethical and practical guidelines, concerns related to ASD, PGT, and abortion decisions may not be alleviated in Taiwan. The development of government policies and regulations is therefore recommended to monitor the delivery, quality, and accuracy of PGT for detecting ASD susceptibility genes. It is also important for professional associations in Taiwan to develop practice guidelines to provide guidance and resources for obstetricians. For example, parents overall have low genetic literacy. Thus, they may have limited capabilities to understand the results of PGT and make informed decisions related to PGT and abortion. Obstetricians may need to explain the testing limitations and diagnosis yields, offer necessary genetic counseling referrals, and provide essential education and explanation to ensure informed decision-making for pregnant women. Additional ethical guidelines should be developed in Taiwan as well. 

With regards to the socio-demographic variables, age was found to be associated with abortion decision-making in this study. In comparison to younger mothers, older mothers in our sample were less likely to terminate the pregnancy if ASD susceptibility genes were found. This finding could possibly be associated with the higher financial stability and lower chance of conception among older mothers. Furthermore, compared to mothers with no religion, those with religious beliefs were less likely to abort pregnancies affected by ASD. This finding is in line with previous research, which suggested that religion may play a role in the intention to undergo PGT and subsequent abortion decision-making among Chinese Americans [20] as well as U.S. and Taiwanese parents of children with ASD [13,19].

Of note, subjective norms related to professionals, which comprised of physicians and other health professions (e.g., nurses, social workers, psychologists, and occupational/physical/speech therapists), were found to be significantly and positively associated with participating mothers’ attitudes toward PGT for detecting ASD susceptibility genes. This result is consistent with past studies, which suggested that individuals’ attitudes toward genetic testing were influenced by health professionals [32,50,52]. Yet, it is important to highlight that the attitudes of the school teachers of the children with ASD could also affect mothers’ attitudes. Such a significant association is in line with previous research, which found that parents might seek ASD-related genetics information from non-health professionals, especially school teachers who have close interactions with children with ASD [38]. Therefore, health professionals must be aware of this issue when communicating with mothers about PGT and may need to provide public education and outreach for Taiwanese school teachers regarding ASD-related genetics/genomics.

Interestingly, the perception of having another child with ASD (i.e., perceived recurrence risk) was not significantly associated with mothers’ attitudes toward PGT for detecting ASD susceptibility genes for their fetuses in this study. The reason may be due to the measurement issue. Participating mothers were asked to use percentages to report how much they believed their next child would have ASD. This question, however, might not have fully captured their beliefs about the recurrence risk. Participants might have different interpretations of recurrence risks with the same percentage. They might also have a limited ability to use a probability approach (i.e., percentages) to estimate their recurrence risk [33]. Utilizing other forms of measures for perceived recurrence risk, such as qualitative measures and the relative (comparable) recurrence risk index, is recommended for future studies. 

This study exhibited some limitations. First, in line with the restrictions of survey research (e.g., inability to survey the entire population and to obtain a 100% response rate) [25], our findings may not be generalized to all Taiwanese mothers of children with ASD. Second, the findings of this study are limited to hypothetical scenarios. The mothers’ actual decisions pertaining to PGT for detecting ASD susceptibility genes and abortion may be different in a real-world setting. Yet, the theoretical construct—intention—aims to capture the participants’ likelihood of undergoing PGT and terminating the affected pregnancies in the near future. Although the participating mothers were asked hypothetical questions, our findings might still strongly predict their actual behavior. Third, although the similar term “PGT for detecting ASD susceptibility genes” has been used in past studies [13,19], there is no formal and consistent name for this testing in Taiwan. Researchers and health professionals may use different terms to refer to the same testing. Lastly, our integrated theoretical framework explained 63.0% of the abortion decision-making among participating mothers of children with ASD. Despite the high percentage, other internal factors (e.g., gestational age for undergoing the PGT, the gender of the fetus in the next pregnancy, future plans to have another child, and psychological discomfort, such as anxiety, worries, and stress) and external factors (e.g., social support as well as awareness of and access to relevant resources) may need to be examined in future studies.

## 5. Conclusions

To the best of our knowledge, this study is the first to assess the psychosocial factors that influence the translation of genomic technologies through the lens of social science theories in Taiwan. Our findings add to the understanding of the views regarding PGT for detecting ASD susceptibility genes within fetuses and subsequent abortion decision-making among a large sample of Taiwanese mothers of children with ASD. This study also represents an initial window to explore the potential impacts of PGT for detecting ASD susceptibility genes on Taiwanese society, addresses the disparities and gaps in this area of research as well as the ethical, legal, and social implications of PGT, and provides insights into better practice and services. As most of the survey respondents favored PGT for detecting ASD susceptibility genes and the termination of ASD-affected pregnancies, actions should be taken to ensure that policies, regulations, and ethical guidelines are established to guide PGT services in Taiwan. Furthermore, the findings and implications of this study are potentially applicable to countries heavily influenced by Chinese culture and areas with Asian immigrants. Researchers and health professionals in Western countries with such PGT services or research available [19,53] can also learn from the experience gained in this study and conduct relevant research to understand the impacts of PGT on families affected by ASD in their countries. 

## Figures and Tables

**Table 1:** Sample characteristics.

Variable	M (SD)/%
Age	38.93 (4.88)
Education level	
Below college	67.9%
College or above	32.1%
Marital status	
Married	90.1%
Others	9.9%
Annual household income	
Less than NT$600,000 (~US$19,745)	35.3%
NT$600,000–1,200,000 (~US$19,745–39,489)	41.0%
Above NT$1,200,000 (~US$39,489)	23.7%
Employment status	
Employed	55.1%
Not employed	44.9%
Religion	
Folk religion	27.6%
Buddhism	27.6%
Christianity	10.0%
No religion	22.4%
Multiple religions	12.4%
Previous abortion experience	
Yes	42.9%
No	57.1%
Family history of ASD	
Yes	35.7%
No	64.3%
Severity level of the child(ren) with ASD ^a^	1.58 (0.76)
Current age of child(ren) with ASD	9.61 (2.13)
Knowledge of PGT for detecting ASD susceptibility genes ^b^	0.33 (0.22)
Perceived genetic etiology of ASD	
Yes	43.4%
No	56.6%

*M*, mean; *SD*, standard deviation; *%*, percentage; *NT$*, New Taiwan Dollars; *~US$*, conversion to United States Dollars; *ASD*, Autism Spectrum Disorders. ^a^ In Taiwan, the severity level of children’s ASD is categorized as mild, moderate, severe, or profound [35,36]. The minimum and maximum severity scores were 1 (i.e., mild) and 4 (i.e., profound), respectively. In this study, 170 children (48.9%) were categorized as mild, 125 children (35.9%) were diagnosed with a moderate level, 44 children (12.6%) were in the severe category, and 9 children’s (2.6%) severity level was profound. ^b^ The knowledge of PGT for detecting ASD susceptibility genes was assessed using seven multiple-choice questions, and the scores were computed utilizing the sum of correct responses divided by seven. The minimum and maximum knowledge scores were 0 and 1, respectively.

**Table 2:** Description, reliability, validity, and example questions of the psychosocial constructs measured in the survey.

Table. *Cont.*	Definition	No. of Items	Example Question	M ^a^/%	SD ^a^	Min ^a^	Max ^a^	TheoreticalRange ^b^	Reliability: Cronbach’s α ^c^	Validity: CFA ^d^
Perceived severity of ASD									α _Overall_ = 0.89	χ^2^ = 51.85, df = 19, *p* < 0.001, RMSEA = 0.072, CFI = 0.976, SRMR = 0.032
Parent-wise perceived severity	Beliefs regarding the seriousness of ASD and its negative impacts on parents’ lives	4	Treatment and education regarding ASD cause extra financial burden on the caregivers [on a 4-point scale ranging from strongly disagree to strongly agree]	3.38	0.54	1.25	4.00	1–4	α = 0.88	
ASD-child-wise perceived severity	Beliefs regarding the seriousness of ASD and its negative impacts on children with ASD	4	ASD affects social life of the children with ASD [on a 4-point scale ranging from strongly disagree to strongly agree]	3.28	0.51	1.50	4.00	1–4	α = 0.81	
Perceived benefits of PGT for detecting ASD susceptibility genes within a fetus									α _Overall_ = 0.93	χ^2^ = 121.49, df = 39, *p* < 0.001, RMSEA = 0.080, CFI = 0.971, SRMR = 0.037
General benefits	Beliefs regarding the helpfulness of PGT for detecting ASD susceptibility genes overall	6	ASD genetic testing is helpful in early treatment and utilization of relevant resources [on a 4-point scale ranging from strongly disagree to strongly agree]	3.19	0.46	2.00	4.00	1–4	α = 0.91	
Family planning-related benefits	Beliefs regarding the helpfulness of PGT for detecting ASD susceptibility genes in family planning	5	ASD genetic testing might be helpful in family planning for parents of children with ASD [on a 4-point scale ranging from strongly disagree to strongly agree]	2.96	0.56	1.00	4.00	1–4	α = 0.91	
Perceived barriers to undergoing PGT for detecting ASD susceptibility genes within a fetus									α _Overall_ = 0.83	χ^2^ = 63.14, df = 24, *p* < 0.001, RMSEA = 0.071, CFI = 0.965, SRMR = 0.043
Testing-related barriers	Beliefs regarding testing related obstacles in undergoing PGT for detecting ASD susceptibility genes	5	The process of undergoing ASD genetic testing is uncomfortable [on a 4-point scale ranging from strongly disagree to strongly agree]	2.58	0.47	1.00	4.00	1–4	α = 0.70	
Social discrimination barriers	Beliefs regarding the prejudice and discrimination related obstacles in undergoing PGT for detecting ASD susceptibility genes	4	The testing results might lead to discrimination against people with ASD [on a 4-point scale ranging from strongly disagree to strongly agree]	2.66	0.60	1.00	4.00	1–4	α = 0.88	
Subjective norms related to PGT for detecting ASD susceptibility genes within a fetus									α _Overall_ = 0.91	χ^2^ = 161.07, df = 42, *p* < 0.001, RMSEA = 0.093, CFI = 0.960, SRMR = 0.057
Professionals	Views and influence of physicians, other non-physician health professionals (e.g., nurses, social workers, occupational/physical/speech therapists, and psychologists), and school teachers on the uptake decision-making of PGT for detecting ASD susceptibility genes	3	If you were pregnant, physicians would recommend PGT for detecting ASD susceptibility genes within your baby [on a 4-point scale ranging from very unlikely to very likely]	9.23	2.87	1.67	16.00	1–16	α = 0.82	
Family members	Views and influence of spouse, spouse’s biological family, participants’ own biological family, and their children without ASD on the uptake decision-making of PGT for detecting ASD susceptibility genes	4	If you were pregnant, your spouse would suggest you undergo PGT for detecting ASD susceptibility genes within your baby [on a 4-point scale ranging from very unlikely to very likely]	6.31	2.69	1.00	16.00	1–16	α = 0.92	
Other people	Views and influence of friends, neighbors, other parents of children without ASD, and general public on the uptake decision-making of PGT for detecting ASD susceptibility genes	4	If you were pregnant, your friends would suggest you undergo PGT for detecting ASD susceptibility genes within your baby [on a 4-point scale ranging from very unlikely to very likely]	4.33	2.03	1.00	12.00	1–16	α = 0.89	
Attitudes toward PGT for detecting ASD susceptibility genes within the fetus	Beliefs and values about PGT for detecting ASD susceptibility genes	4	All pregnant women should undergo PGT for detecting ASD susceptibility genes within their babies [4-point scales ranging from strongly disagree to strongly agree and from very unimportant to very important]	9.01	3.41	2.25	16.00	1–16	α = 0.83	χ^2^ = 4.70, df = 2, *p* = 0.095, RMSEA = 0.067, CFI = 0.994, SRMR = 0.015
Self-efficacy in undergoing PGT for detecting ASD susceptibility genes within the fetus	Confidence in going through PGT for detecting ASD susceptibility genes	4	If you were pregnant, considering the factor of time, on a scale of 0 to 10, how confident are you in undergoing PGT for detecting ASD susceptibility genes within your baby? [11-point scale ranging from 0 to 10]	6.13	3.11	0	10.00	0–10	α = 0.93	χ^2^ = 9.57, df = 3, *p* = 0.023, RMSEA = 0.082, CFI = 0.995, SRMR = 0.012
Perceived recurrence risk of having another child with ASD^e^	Beliefs regarding the chance of having another child with ASD	1	Suppose you plan to have another child; the chance of having another child with ASD is ______ % [0-100% (0% = child will not have ASD; 100% = child will definitely have ASD)]	36.53	27.68	0	100.00	0–100		
Intention to undergo PGT for detecting ASD susceptibility genes within the fetus ^e^	Likelihood of undertaking PGT for detecting ASD susceptibility genes in the future	1	If you were pregnant, how likely would you to undergo PGT for detecting ASD susceptibility genes within your baby? [4-point scale ranging from very unlikely to very likely]	2.89	0.83	1.00	4.00	1–4		
Intention to terminate ASD-affected pregnancies^e^	Likelihood in decision regarding continuation or termination of ASD-affected pregnancies in the future	1	If PGT results indicate that you might have a child with ASD, what would be your choice? [give birth to the child or do not keep the child (abortion)]	Give birth to the child: 46.9% Do not keep the child (abortion): 53.1%						

*No.*, number; *M*, mean; *%*, percentage; *SD*, standard deviation; *Min*, minimum; *Max*, maximum; *α*, alpha; *CFA*, confirmatory factor analysis; *ASD*, autism spectrum disorder; *χ^2^*, chi-square; *df*, degrees of freedom; *RMSEA*, root mean square error of approximation; *CFI*, comparative fit index; *SRMR*, standardized root mean square residual. ^a^ Descriptive statistics of the collected survey data for each of the whole scale/subscale. ^b^ Possible range of scores for each of the whole scale/subscale. ^c^ An acceptable reliability was considered for those with a Cronbach’s α equal or greater than 0.70 [49].^d^ An acceptable construct validity was considered for those with a RMSEA smaller than 0.10, CFI greater than 0.90, and SRMR smaller than 0.08 [47]. ^e^ Cronbach’s alpha and confirmation factor analysis could not be conducted as it was a single-item measure.
